# Two new species of *Sabulina* (Caryophyllaceae) from Washington State, U.S.A.

**DOI:** 10.3897/phytokeys.81.13106

**Published:** 2017-06-15

**Authors:** Ben S. Legler, Markus S. Dillenberger

**Affiliations:** 1 WTU Herbarium, Burke Museum, Box 355325, University of Washington, Seattle, WA 98195-5325, U.S.A.; 2 Department of Botany and Plant Pathology, Oregon State University, 2082 Cordley Hall, Corvallis, OR 97331, U.S.A.

**Keywords:** Caryophyllaceae, *Arenaria*, *Minuartia*, *Sabulina*, Washington, Olympic Mountains, Twin Sisters, endemic, new species

## Abstract

*Sabulina basaltica* and *Sabulina sororia* (Caryophyllaceae) are described as new species endemic to Washington State, U.S.A. *Sabulina basaltica* is restricted to high-elevation, basalt rocks in the northeastern Olympic Mountains, and *Sabulina sororia* to high-elevation, dunite rocks of the Twin Sisters Range in the North Cascade Mountains. Both were previously confused with *Sabulina rossii* (formerly called *Arenaria rossii* or *Minuartia rossii*). Their recognition as distinct species is supported by morphological and molecular characters and disjunct geographic distributions. Both are illustrated, mapped and compared to related species. We also present a molecular phylogeny of *Sabulina* based on nuclear ITS and plastid trnQ-rps16 DNA with increased sampling of North American taxa. The phylogeny resolves a single clade containing all glabrous, perennial, North American *Sabulina* taxa including *Sabulina rossii* and both of the new species.

## Introduction

While preparing a new Flora of the Pacific Northwest (Giblin et al., in press) the status of specimens from the North Cascade and Olympic Mountains in Washington State formerly called Arenaria rossii R. Br. ex Richardson var. rossii (Hitchcock et al. 1964, Hitchcock and Cronquist 1973) came into question. Ensuing study of the specimens demonstrated they do not fit any currently described species and are furthermore distinct from each other. On the basis of recent field work, morphological analyses, genetic sequencing, and disjunct distributions, they are here described as two new species in order to make names available for use in the new Flora.

Herbarium specimens of the two new species were first collected in 1911 from the Olympic Mountains and in 1939 from the Twin Sisters Range in the North Cascade Mountains, with the most recent collections prior to this study made in 1984 from the Olympic Mountains and in 1968 from the Twin Sisters Range. These specimens, 17 in total, are held by three local herbaria (OLYM, WTU and WWB; acronyms according to Thiers continuously updated); no duplicates were located through searches of digitized specimens at other herbaria.

These specimens have largely been overlooked in previous studies of *Arenaria* L. s. lat. To our knowledge, no floras or literature treat them under any names other than A. rossii var. rossii or *Minuartia rossii* (R. Br. ex Richardson) Graebn., with the exception of a vague reference to *M. stricta* (Sw.) Hiern. by Wolf et al. (1979). Maguire (1958) recognized three subspecies of *A. rossii* with a circumboreal distribution extending south to the U.S. Rocky Mountains but made no mention of plants from Washington. Wolf et al. (1979), following a revision of *Arenaria* by McNeill (1962), treated Maguire’s three subspecies at the rank of species under the segregate genus *Minuartia* Loefl., as *M. austromontana* S.J. Wolf & Packer, *M. elegans* (Cham. & Schltdl.) Schischk., and *M. rossii*. In describing *M. austromontana*, Wolf et al. (1979) examined several of the herbarium specimens fromWTU, concluding that “reports of this species from Washington are erroneous,” and vaguely referred the specimens to *M. stricta* (Sw.) Hiern, a species not otherwise attributed to Washington by any sources. The most recent comprehensive treatment of *Minuartia* for North America (Rabeler et al. 2005) does not account for the Washington specimens and excludes Washington from the distributions of *M. austromontana*, *M. elegans*, *M. rossii*, and *M. stricta*.

Recent phylogenetic studies (Harbaugh et al. 2010, Greenberg and Donoghue 2011, Dillenberger and Kadereit 2014) clearly demonstrate that *Minuartia*, as defined by McNeill (1962) and applied by Rabeler et al. (2005), is highly polyphyletic. Dillenberger and Kadereit (2014) proposed a new generic classification for *Minuartia* s. lat. and resurrected the segregate genus *Sabulina* Rchb., newly circumscribed to include ca. 65 taxa widely distributed throughout the Northern Hemisphere. Of the 33 North American taxa formerly placed under *Minuartia* s.l. by Rabeler et al. (2005), 19 now belong to *Sabulina*, including *S. austromontana* (S.J. Wolf & Packer) Dillenb. & Kadereit, *S. dawsonensis* (Britton) Rydb., *S. elegans* (Cham. & Schltdl.) Dillenb. & Kadereit, *S. macrantha* (Rydb.) Dillenb. & Kadereit, *S. michauxii* (Fenzl) Dillenb. & Kadereit, *S. rossii* (R. Br. ex Richardson) Dillenb. & Kadereit, and *S. stricta* (Sw.) Rchb. These taxa together are informally referred to here as the *S. rossii* species complex. Of these taxa, only one sample of *S. stricta* and one sample misidentified as *S. dawsonensis* (see Suppl. material 2) were included in the phylogeny by Dillenberger and Kadereit (2014); assignment of the remaining taxa was inferred by morphology. Morphological similarities likewise suggest the two new species from Washington belong to *Sabulina*. To confirm their placement and to clarify relationships between the new species and the above taxa, we present an expanded phylogeny of *Sabulina*.

## Methods

A total of 127 herbarium specimens from ALA, KHD, MONTU, OLYM, UBC, V, and WTU for the above taxa were physically examined for morphological characters (see Suppl. material 1). Digital images of additional herbarium specimens accessible online (e.g., CPNWH 2017) from ALA, MONT, MONTU, WWB, and YU were examined for macromorphological characters. Identifications were verified for all specimens examined. Comparative measurements given below (e.g., Table 1) were obtained from herbarium specimens and published literature sources (e.g., Rabeler et al. 2005). Geographic distributions of related species were obtained from published literature sources and verifiable herbarium specimens. Field work focused on visiting known locations, with visits to the Olympic Mountains July 15–16 and July 24, 2016, and to the Twin Sisters Range August 6–7, 2016, to collect additional herbarium specimens, tissue samples for DNA extraction, and obtain information about distribution, habitat, and plant morphology. Living plants were photographed using a Nikon D800 digital SLR camera with 50 mm macro lens. Measurements for the two new species were obtained from living material and dried specimens, with herbarium specimens examined at 10×–40× magnification using a dissecting microscope with a calibrated, 0.1 mm scale (at 10×) ocular ruler.

**Table 1.** Morphological comparisons of *Sabulina basaltica*, *S. sororia*, and other glabrous, perennial *Sabulina* species in North America.

Taxon	Growth form	Leaves	Inflorescence	Pedicels	Sepals (at anthesis)	Petals	Capsules	Seeds
***Sabulina austromontana***	cespitose or dense mats, 1–3 cm tall	3−10 mm, 1-veined	flowers solitary	3–15(–20) mm	2–3 mm, narrowly to broadly lanceolate, 3-veined, green	absent or rudimentary (rarely = sepals)	2–3 mm, ca = sepals	0.6–1 mm, brown, obscur-ely rugose
***Sabulina basaltica***	cespitose to tightly mat-forming, 0.5–3 cm tall	(0.6−)1−3.5(−4.5) mm, 3-veined	2–5(–8)-flowered cymes, with some flowers solitary	1−3.5(−6) mm	(1.6−)2.4−2.8(−3.3) mm, lanceolate to narrowly ovate-lanceolate, 3-veined, light green	(1−)1.2−1.8(−2) × as long as sepals	1.8–2.4 mm, < or = sepals	0.6–0.8 mm, dark reddish-brown to blackish, lightly rugose
***Sabulina dawsonensis***	loosely cespi-tose, 4–30 cm tall	4−15 mm, 1-veined to weakly 3-veined	(2–)7–15-flowered cymes	3–25 mm	2.5–4 mm, ovate to broadly lanceolate, 3-veined, green to purplish	0.5–0.8 × as long as sepals	3.5–4.5 mm, > sepals	0.5–0.6 mm, dark brown to blackish, lightly rugose
***Sabulina elegans***	loosely cespi-tose, 3–8 cm tall	3−10 mm, 1-veined	flowers solitary	10–40 mm	2–4 mm, ovate to lanceolate, 3-veined, purplish	0.6–1(–1.1) × as long as sepals (rarely absent)	2–4 mm, ca = sepals	0.6–1 mm, reddish-brown, lightly rugose
***Sabulina macrantha***	mat-forming to trailing, 2–15 cm tall	5–10 mm, 1-veined to weakly 3-veined	2–5(–8)-flowered cymes or some flowers solitary	2–15(–20) mm	3.5–5 mm, ovate to lanceolate, 3-veined, green to purplish	0.7–1.8 × as long as sepals	3–3.8 mm, < sepals	0.7–1.1 mm, blackish, distinctly rugose
***Sabulina michauxii***	loosely cespitose (occ. matted), 8–40 cm tall	8–30 mm, 1–3-veined	5–30-flowered	3–60 mm	3–6 mm, ovate to lanceolate, 3-veined, green	1.3–2 × as long as sepals, or < sepals in northern plants	3–4 mm, usually < sepals	0.8–0.9 mm, blackish, prominently rugose
***Sabulina rossii***	pulvinate to cespitose, 1–3 cm tall	1–4 mm, 1-veined	flowers solitary	1–20 mm	1.5–2.5 mm, oblong-ovate, 1-veined, purplish	1.2–2 × as long as sepals, or < sepals, or absent	1.5–2.5 mm, ca = sepals	0.6 mm, brown, obscurely rugose
***Sabulina sororia***	mat-forming to trailing, 0.5–4 cm tall	1.2−3.5(−5) mm, 1-veined	2–3-flowered cymes with some flowers solitary	(1−)2−8(−15) mm	(1.4−)1.7−2.5(−3) mm, ovate-lanceolate, 3-veined, green to purplish-tinged	1.3–2(–2.5) × as long as sepals	1.8–2.6 mm, > or rarely = sepals	0.7–0.8 mm, reddish-black, lightly rugose
***Sabulina stricta***	cespitose or mat-forming, 0.8–12 cm tall	2.5–14 mm, 1-veined to weakly 3-veined	2–3(–5)-flowered cymes or some (rarely all) flowers solitary	1–35 mm	(1.5–)2–3.5 mm, elliptic to ovate-lanceolate, 3-veined, green to purplish	0.6–1 × as long as sepals, or rudimentary to absent	2.5–3.2 mm, < or = sepals	0.4–0.6 mm, reddish–brown, obscurely rugose

For the molecular phylogeny, we used 14 samples of the *S. rossii* species complex including three samples in total of the two new species (see Suppl. material 2). Total genomic DNA was extracted using the FastDNA Kit (MP Biomedicals, Santa Ana, CA, U.S.A.) following the manufacturer’s protocol, but adding 40 µl 1% polyvinylpyrrolidone during cell lysis. PCRs of nuclear internal transcribed spacer (ITS) and plastid spacer trnQ-rps16 were carried out with One*Taq* 2x Master Mix (New England Biolabs, Ipswich, MA, U.S.A.) following the recommendations for reaction mix and PCR cycle program. For ITS, primers ITS4 and 5 (White et al. 1990) were used; for trnQ-rps16, trnQ^(UUG)^ and rps16x1 (Shaw et al. 2007). Annealing temperature for ITS was 52° C, for trnQ-rps16 56° C. PCR products were cleaned up with DNA Clean & Concentrator-5 Kit (Zymo Research, Irvine, CA, U.S.A.), following the manufacturer’s protocol. Cycle sequencing was carried out with the same primers as the PCR. Sequencing was carried out on an ABI 3730 capillary sequence machine at the Center for Genome Research and Biocomputing at Oregon State University. Sequencher v.4.10.1 (Gene Codes, Ann Arbor, Michigan, U.S.A.) was used for trace file editing, and sequences were submitted to GenBank (see Suppl. material 2).

Sequences were aligned using MUSCLE v.3.8.31 (Edgar 2004) implemented in seaview v.4.3.0 (Gouy et al. 2010). Maximum likelihood phylogenies were obtained using RAxML v.8.0.26 (Stamatakis et al. 2008) with the GTR+Γ substitution model and the fast bootstrap algorithm with automatic halt based on the autoMRE criterion. Sequences of ITS and trnQ-rps16 were analysed separately, taxon sampling was complemented with published sequences from GenBank for *Sabulina michauxii* and *S. fontinalis*, and previously sequenced but unpublished sequences of *Sabulina* (see Suppl. material 2).

## Results

Morphological comparisons indicate the plants from the Olympic Mountains (*Sabulina basaltica* in Table 1 and the key) differ from plants from the Twin Sisters Range (*S. sororia*) in leaf veination, sepal shape, sepal length, sepal length:width ratio, and capsule length relative to sepals, with minimal or no overlap between the two species in these characters (Table 1). The plants show additional, though more strongly overlapping differences in growth form, pedicel length, and stem, leaf, and sepal color. The Olympic Mountains plants and Twin Sisters plants together differ from all other glabrous, perennial *Sabulina* taxa in North America by the combination of partially cymose inflorescences, petals 1.2–2.5 times as long as the sepals, sepals 1.5–2.8(–3.3) mm, capsules 1.8–2.6 mm, and dark reddish-brown to blackish seeds 0.6–0.8 mm.

Geographically, the Olympic Mountain plants are separated from the Twin Sisters plants by a distance of ca. 130 air km across the Puget Sound trough (Fig. 6). The nearest known populations of other glabrous, perennial *Sabulina* species are in northeast Oregon (*S. austromontana*) at a distance of ca. 520 air km and in the Rocky Mountains of southern Canada (*S. austromontana* and *S. dawsonensis*) at a distance of ca. 640 air km.

**Figure 1.**
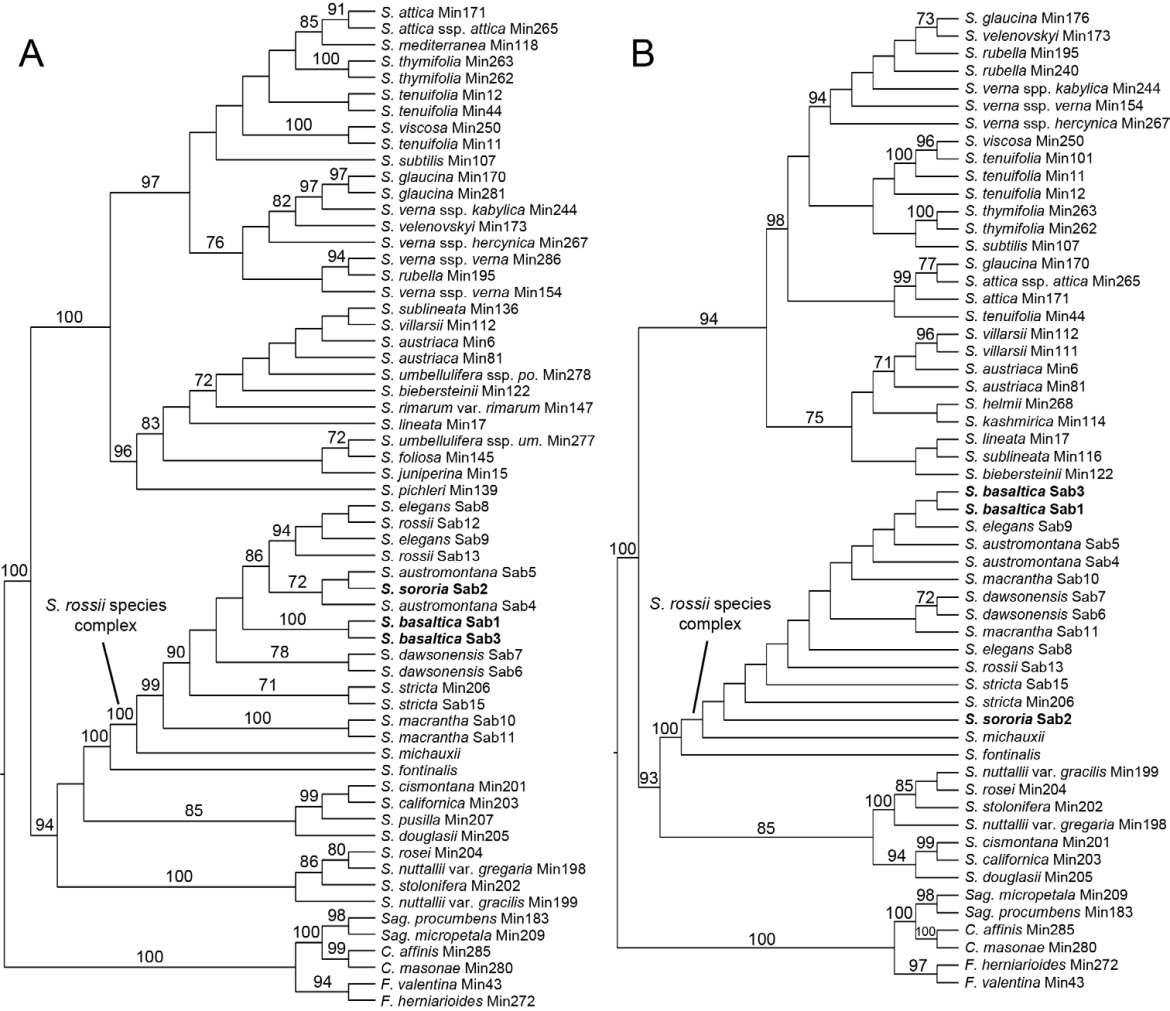
Maximum likelihood phylogenies of *Sabulina*. **A** Cladogram of the ITS dataset **B** Cladogram of the trnQ-rps16 dataset. Phylogenies obtained with RAxML, values above branches are bootstrap support values (only ≥ 70 shown) *C.*, *Colobanthus*; *F.*, *Facchinia*; *S.*, *Sabulina*; *Sag.*, *Sagina*.

The molecular phylogeny of the ITS data set (Fig. 1A) shows that the *S. rossii* species complex is monophyletic and highly supported (bootstrap support (BS) 100), and is sister to *S. fontinalis* (Short & R. Peter) Dillenb. & Kadereit (BS 100). Relationships among members of the *S. rossii* species complex were not fully resolved, but *S. basaltica* (BS 100), *S. dawsonensis* (BS 78), *S. stricta* (BS 71) and *S. macrantha* (BS 100) are supported to be monophyletic. *Sabulina austromontana* is closely related to *S. sororia* (BS 72). *Sabulina rossii* and *S. elegans* form a monophyletic group (BS 94) without support for the species within. *Sabulina basaltica* is part of a polytomy with *S. dawsonensis* and *S. stricta* and a clade comprising *S. rossii*, *S. elegans*, *S. austromontana* and *S. sororia*. The phylogeny of the plastid trnQ-rps16 marker (Fig. 1B) is less resolved, but the *S. rossii* species complex is also supported with a BS of 72. Only *S. dawsonensis* is supported as monophyletic (BS 83).

### Taxonomic treatment

#### Sabulina basaltica

Taxon classificationPlantaeCaryophyllalesCaryophyllaceae

B.S.Legler sp. nov.

urn:lsid:ipni.org:names:60474719-2

Figs 2A –E , 3

##### Type.

U.S.A. Washington, Clallam Co.: Olympic National Park: along climbers trail at base of summit block on west side of Mt. Angeles, 1872 m, 47.995079°N, 123.468522°W, 15 Jul 2016, *B.S. Legler 14177* (holotype: WTU!; isotype: OLYM!).

##### Diagnosis.

Differs from all other glabrous, perennial *Sabulina* species in North America by the combination of 3-veined dried leaves, flowers partly in 2–5(–8)-flowered cymes, sepals mostly < 3 mm long, petals conspicuously longer than the sepals, capsules 1.8–2.4 mm long and mostly < or = sepals, and dark reddish-brown to reddish-black seeds 0.6–0.8 mm long.

##### Description.

*Plants* perennial, forming dense (rarely loose) mats or cushions 2–8(–12) cm diameter, glabrous throughout. *Taproot* slender to slightly thickened, 1–3 mm diameter near summit. *Stems* numerous, radially spreading from the taproot, prolifically branching; older stems decumbent to ascending, 1–6 cm, brown to tan; new shoots arising from axillary fascicles on previous year’s stems, ascending to erect, 0.5–3 cm, internodes of flowering shoots 0.1–1(–2) times as long as leaves, light green or maroon-tinged. *Leaves* usually strongly overlapping, occasionally well-spaced, connate proximally to form a tight, scarious sheath; blade (0.6–)1–3.5(–4.5) × 0.3–0.6 mm, ascending to nearly appressed, straight to slightly incurved or slightly recurved, light green to yellowish-green, not or only weakly shiny, subulate, rounded abaxially, nearly flat adaxially, veins not visible in fresh material, margins rounded, not scarious, smooth, apex obtuse to rounded, usually maroon; axillary fascicles of leaves usually present; previous year’s leaves marcescent, long-persistent on older stems, with the midvein and two lateral veins becoming prominent, rigid. *Inflorescences* terminal, 2–5(–8)-flowered, open cymes usually mixed with solitary terminal flowers; bracts 1.1–2.6 mm, subulate to lanceolate, incurved, green with scarious margins, rounded abaxially, flat to concave adaxially, apex obtuse to bluntly acute. *Pedicels* 1–3.5(–6) mm, glabrous. *Flowers* perfect or functionally male or functionally female, many plants functionally monoecious to nearly dioecious. *Hypanthium* obscure, disc-shaped. *Sepals* spreading-ascending at anthesis, light green, glabrous, lanceolate to narrowly ovate-lanceolate, (1.6–)2.4–2.8(–3.3) × 0.7–0.9(–1.1) mm, (2.4–)3–3.2(–3.5) times as long as wide, scarious margins ca. 0.05–0.2 mm wide, base cupped, apex green to maroon, acute to shortly acuminate, outer surface flat to convex, weakly 3-veined at anthesis, becoming distinctly 3-veined in fruit or when dried. *Petals* white, spreading, narrowly to broadly oblong or narrowly obovate, 3.2–5.2 × 1.1–2 mm, (1–)1.2–1.8(–2) times as long as sepals, base gradually tapered to a short, greenish-yellow claw, apex rounded to truncate, entire to weakly erose or slightly emarginate. *Nectaries* 5, at base of outer stamens, greenish-yellow, ca. 0.4 × 0.4 mm, truncate, alternate with the petals. *Stamens* 10, in 2 series of 5, either all fertile or all abortive; filaments subulate, whitish-green; anthers orbiculate, pale yellow; fertile stamens with filaments 1.4–2.5 mm and anthers 0.4–0.5 mm; abortive stamens with filaments 0.2–0.5 mm and anthers 0.1–0.2 mm. *Ovary* superior; placentation shortly free-central; ovules usually 12 per ovary. *Styles* 3, distinct, erect to ascending; functionally male flowers with styles ca. 0.7 mm and stigmas scarcely developed; functionally female flowers with styles 1–1.7 mm and stigmas linear, glandular-puberulent adaxially. *Capsules* light green to greenish-tan (valve margins tan), on stipe ca. 0.1–0.2 mm, ovoid-conical, 1.8–2.4 × 1–1.5 mm, slightly shorter than or equaling (rarely slightly longer than) and usually enclosed by the appressed sepals and withering-persistent petals, dehiscing in upper half by 3 valves, these becoming incurved on margins and slightly recurved at tip. *Seeds* 4–8 per capsule, 0.6–0.8 mm, dark reddish-brown to reddish-black, obliquely reniform with radicle prolonged into a curved bump, somewhat compressed, with rounded margins, surfaces sculpted with low, rounded, slightly elongate and sinuous bumps at > 10× magnification.

##### Additional specimens examined.

**U.S.A. Washington, Clallam Co.** Third Peak, Mt. Angeles, 10 Aug 1911, *no collector* (OLYM); Mt. Angeles, 5500 ft, 2 Aug 1930, *J.W. Thompson 5481* (WTU); Mt. Angeles, 5500 ft, 10 Jul 1931, *G.N. Jones 3202* (WTU); Mt. Angeles, 6800 ft, 17 Jul 1931, *J.W. Thompson* 7433 (WTU); Mt. Angeles, 5500 ft, 15 Jul 1933, *J.W. Thompson 9458* (WTU); Mt. Angeles, 15 Jul 1933, *H.E. Helmrich 259* (WTU); Mt. Angeles, 12 Jul 1936, *M.P. Harthill s.n.* (OLYM); Mt. Angeles, 31 Jul 1966, *L.C. Bliss* s.n. (WTU); Saddle between Mt. Baldy and Mt. Tyler, 5600 ft, 23 Jul 1976, *N. Buckingham 514* (OLYM); Blue Mountain, northeast ridge, T28N R5W S1, 5600 ft, 31 Jul 1984, *E.L. Tisch 2724* (OLYM); Blue Mountain, northeast ridge, T28N R5W S1, 5600 ft, 31 Jul 1984, *E.L. Tisch 2724 1/2* (OLYM); Along ridgeline ca. 100 meters southwest of summit of Mt. Angeles, 47.994735°N, 123.467501°W; 1896 m, 15 Jul 2016, *B.S. Legler 14178* (WTU); High point at east end of ridgeline along summit of Mt. Angeles, 47.995365°N, 123.463590°W; 1949 m, 15 Jul 2016, *B.S. Legler 14179* (WTU); Southeast rib of Steeple Rock along Hurricane Ridge, 47.961464°N, 123.452969°W; 1657 m, 16 Jul 2016, *B.S. Legler 14183* (WTU); South side of summit of Eagle Point, along Hurricane Ridge, 47.938951°N, 123.409042°W; 1893 m, 16 Jul 2016, *B.S. Legler 14184* (WTU, OLYM); **Jefferson Co.**: Iron Mountain, 6000 ft., 21 Jul 1934, *J.W. Thompson* 11054 (WTU); Ridge north from Buckhorn Pass, T27N R4W S13, 6600 ft, 1 Aug 1981, *N. Buckingham 2658* (OLYM); West face of Buckhorn Mountain just above ridgeline that connects Buckhorn Mountain to Peak 6988, 47.826286°N, 123.117615°W; 2026 m, 24 Jul 2016, *B.S. Legler 14195* (WTU).

##### Etymology.

The epithet *basaltica* refers to the basalt rock to which this species is apparently restricted.

##### Vernacular name.

Suitable common names are Olympic sandwort or basalt sandwort.

##### Distribution and ecology.

*Sabulina basaltica* is known only from subalpine and alpine peaks along the northeastern rim of the Olympic Mountains in Clallam and Jefferson counties, Washington, U.S.A. (Fig. 6C), at documented elevations of 1650–2100 meters. It is presently known from seven peaks: Mt. Angeles, Steeple Rock, Eagle Point, Blue Mountain, near Mt. Tyler, Buckhorn Mountain, and Iron Mountain. It is apparently confined to south or southwest facing rock faces composed of ocean floor basalts (mainly pillows and breccia) of the Crescent Formation (Tabor and Cady 1978, Babcock et al. 1992) on ca. 30–60° slopes (Fig. 5A). *Sabulina basaltica* occur as scattered individuals forming small tufts in exposed rock crevices with very sparse vascular plant cover (Fig. 5B–C). The rock faces are exposed to solar radiation and wind. Snow accumulation is likely minimal due to wind ablation, and meltout likely occurs much earlier than on adjacent slopes. No plants were found on more protected east or north facing slopes, nor on more gentle slopes around the periphery of rock faces, whether vegetated or not.

Directly associated species include *Anemone multifida* Poir., Antennaria cf. rosea Greene, *Campanula piperi* Howell, *Carex nardina* Fr., *Dasiphora fruticosa* (L.) Rydb., *Erigeron compositus* Pursh, Penstemon davidsonii Greene var. menziesii (D.D. Keck) Cronquist, *Petrophytum hendersonii* (Canby) Rydb., *Phlox diffusa* Benth., Polemonium pulcherrimum Hook. subsp. pulcherrimum, *Potentilla villosa* Pall. ex Pursh, *Sabulina rubella* (Wahlenb.) Dillenb. & Kadereit, *Salix nivalis* Hook., *Saxifraga austromontana* Wiegand, *Saxifraga cespitosa* L., *Sedum lanceolatum* Torr., *Selaginella wallacei* Hieron., *Smelowskia americana* Rydb., *Trisetum spicatum* (L.) K. Richt., and *Viola flettii* Piper. Crustose lichens cover most of the rock surfaces. Adjacent conifer species at subalpine sites include *Callitropsis nootkatensis* (D. Don) D.P. Little, *Abies lasiocarpa* (Hook.) Nutt., Juniperus communis L. var. kelleyi R.P. Adams, and *Pinus albicaulis* Engelm.

Although oceanic basalts form an extensive belt around the northern, eastern, and southeastern sides of the Olympic Mountains (Tabor and Cady 1978), suitable climatic conditions for *S. basaltica* presumably occur only in the northeastern portion of the mountains within a rain shadow formed by one of the steepest precipitation gradients in North America (Phillips and Donaldson 1972). Average annual precipitation levels for Mt. Angeles and Buckhorn Mountain are estimated at ca. 200 cm (PRISM 2017). For comparison, Mt. Olympus, only 28 km to the southwest of Mt. Angeles, receives an estimated 600 cm of precipitation annually and Sequim, 30 km to the northeast, only 41 cm of annual precipitation (PRISM 2017). Furthermore, the growing season is relatively dry, with ca. 12% of total annual precipitation falling during May–September at Mt. Angeles (PRISM 2017). Based on climate and substrate, areas of suitable habitat for *S. basaltica* are prediced to occur in a discontinuous arc extending from the vicinity of Hurricane Ridge in the north to at least the vicinity of Mt. Constance to the southeast, and possibly farther south to The Brothers and adjacent peaks (Fig. 6C). Basalts of the Crescent Formation reappear on the southern tip of Vancouver Island, British Columbia, just to the north of the Olympic Mountains (Babcock et al. 1992), though at much lower elevations where previously covered by the Cordilleran ice sheet; we do not expect *S. basaltica* to occur there.

A pair of specimens from Blue Mountain, Clallam County (*E. L. Tisch 2724* and *E. L. Tisch 2724 1/2*, OLYM) indicate on the label that plants were collected from “crevices in (limestone?) rock outcrop,” raising the possibility that *S. basaltica* is not confined to basalt. However, Blue Mountain contains outcrops of basalt rocks and a return visit to the site would be needed to determine the actual rock type from which the specimens were collected.

##### Phenology.

Specimens of *Sabulina basaltica* with flowers were collected from mid July to mid August, and specimens with fruits from mid July to early August.

##### Conservation status.

Population sizes on Mt. Angeles, Steeple Rock, and Eagle Point were estimated at ca. 1000, 100, and 300 plants, respectively, during visits in 2016. No estimate was attempted at Buckhorn Mountain due to difficulty of access, but about 30 plants were observed in the immediate vicinity of *Legler 14195*. Significant areas of potentially suitable habitat occur on other basalt peaks in the northeastern Olympic Mountains within a predicted area of extent of ca. 50 km^2^; however, the vascular plant flora for the majority of these peaks remains poorly documented or undocumented with herbarium specimens (CPNWH 2017), and the lack of information precludes range-wide estimates of the total number of populations and plants. Assignment of a formal conservation status may require additional field work to gauge rarity. All known populations and nearly all areas of potentially suitable habitat are protected within Olympic National Park and adjacent wilderness areas. The known populations are located on steep rock slopes away from trails and roads. Therefore, direct anthropogenic impacts are assumed to be minimal. Grazing pressure and disturbance from introduced mountain goats (*Oreamnos americanus* Blainville, 1816) pose an increasing impact to high elevation plant communities in the Olympic Mountains (Houston et al. 1994, Jenkins et al. 2012), and goats were observed in the vicinity of populations of *S. basaltica* on Mt. Angeles during the visit in 2016; however, no evidence of direct grazing or damage to *S. basaltica* was detected.

**Figure 2.**
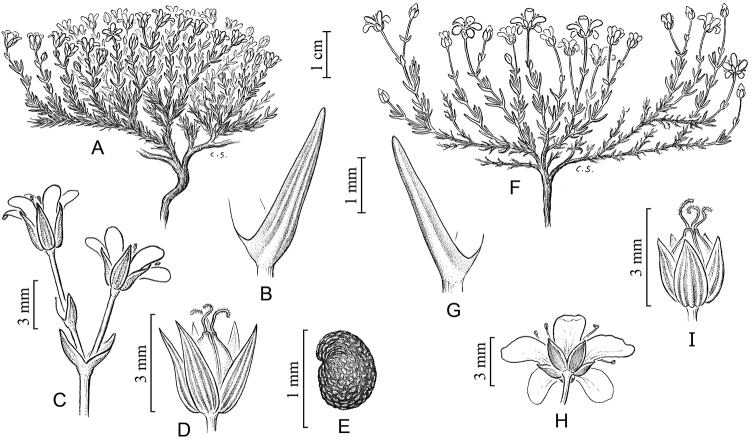
Line drawings of *Sabulina basaltica* and *Sabulina sororia*. A–E. *Sabulina basaltica*. **A** Habit **B** Dried leaf with 3 veins **C** Cymose, bracteate inflorescence with two flowers **D** Capsule with dried, 3-veined sepals (with sepals pushed outwards and withered petals removed to reveal capsule) **E** Seed **F–I** *Sabulina sororia* **F** Habit **G** Dried leaf with 1 vein **H** Flower **I** Capsule with dried, 3-veined sepals (withered petals removed).

**Figure 3.**
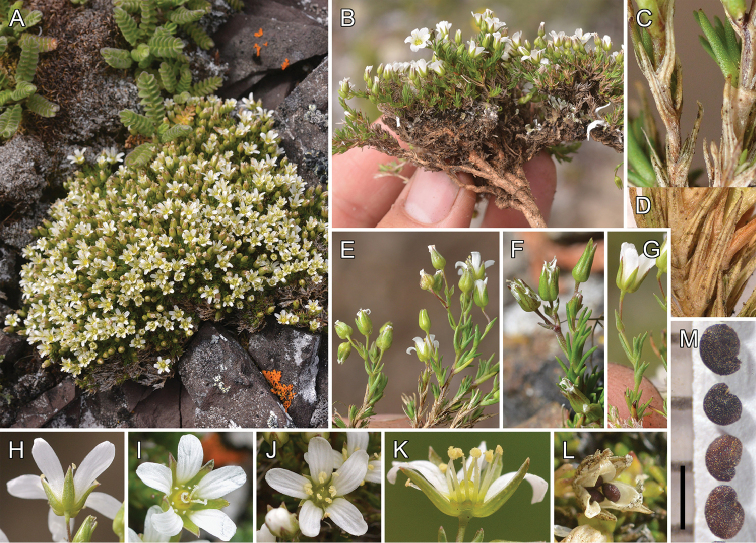
*Sabulina basaltica*. **A** Plant forming a tight mat (*Legler 14178*) **B** Excavated plant with taproot (*Legler 14184*) **C** Fresh leaves and persisting, 3-veined, dead leaves (*Legler 14177*) **D** Dead, persisting, 3-veined leaves (*Legler 14178*) **E–F** Cymose inflorescences (*Legler 14177*, *Legler 14175*) **G** Solitary terminal flower (*Legler 14184*) **H** Sepals and petals, showing shapes and lengths (*Legler 14184*) **I–J** Flowers with different combinations of stamen and style lengths (*Legler 14177*, *Legler 14184*) **K** Partially dissected flower showing hypanthium and nectaries (*Legler 14183*) **L** Dehisced capsule with seeds (*Legler 14183*) **M** Seeds (*Legler 14183*). Black scale bar is 1 mm.

#### Sabulina sororia

Taxon classificationPlantaeCaryophyllalesCaryophyllaceae

B.S.Legler sp. nov.

urn:lsid:ipni.org:names:60474720-2

Figs 2F –I , 4

##### Type.

U.S.A. Washington, Whatcom Co.: Mt. Baker-Snoqualmie National Forest, on west side of ridge along Sisters Divide 0.45 air km southeast of outlet of Lake Wiseman, Twin Sisters Range, 1414 m, 48.707131°N, 121.934086°W, 6 Aug 2016, *B.S. Legler 14263* (holotype: WTU!; isotypes: MICH!, MO!, NY!, UBC!).

##### Diagnosis.

Differs from all other glabrous, perennial *Sabulina* species in North America by the combination of 1-veined dried leaves, flowers partly in 2–3-flowered cymes, sepals mostly < 2.5 mm long, petals conspicuously longer than the sepals, capsules 1.8–2.6 mm long and mostly > sepals, and reddish-black seeds 0.6–0.8 mm long.

##### Description.

*Plants* perennial, forming loose to dense mats 2–20 cm in diameter, glabrous throughout. *Taproot* slender to slightly thickened, 1–3 mm diameter near summit. *Stems* numerous, radially spreading from the taproot, prolifically branching; older stems decumbent to ascending, 1–10 cm, brown to tan; new shoots arising from axillary fascicles on previous year’s stems, ascending to erect, 1–4 cm, internodes of flowering shoots 0.3–2(–3) times as long as leaves, deep green or purplish. *Leaves* slightly to strongly overlapping or well-spaced, connate proximally to form a tight, scarious sheath; blade 1.2–3.5(–5) × 0.4–0.7 mm, ascending to spreading-ascending, straight to slightly incurved or slightly recurved, green to deep green, often maroon-tinged, shiny, subulate, rounded abaxially, nearly flat adaxially, veins not visible in life, margins rounded, not scarious, smooth, apex obtuse to rounded, usually maroon; axillary fascicles of leaves usually present; previous year’s leaves loosely marcescent on older stems, with only the midvein visible and persisting (no lateral veins). *Inflorescences* terminal, 2–3-flowered, open cymes, usually mixed with solitary terminal flowers; bracts 0.7–1.6 mm, lanceolate to ovate-lanceolate, incurved, green or maroon with scarious margins, rounded abaxially, flat to concave adaxially, apex obtuse to bluntly acute. *Pedicels* (1–)2–8(–15) mm, glabrous. *Flowers* perfect or functionally male or functionally female, most plants functionally monoecious to nearly dioecious. *Hypanthium* obscure, disc-shaped. *Sepals* spreading-ascending at anthesis, deep green, often lightly maroon-tinged, glabrous, broadly ovate to ovate-lanceolate, (1.4–)1.7–2.5(–3) × 0.6–1.1(–1.3) mm, 1.5–2.5(–3.5) times as long as wide, scarious margins ca. 0.05–0.15 mm wide, base cupped, apex green to maroon, acute, outer surface convex, smooth to very weakly 3-veined at anthesis, becoming 3-veined in fruit or when dried. *Petals* white, spreading, broadly oblong to obovate, 3.2–4(–5.2) × 1.2–2(–2.6) mm, 1.3–2(–2.5) times as long as sepals, base gradually tapered to a short, greenish-yellow claw, apex rounded to weakly truncate. *Nectaries* 5, at base of outer stamens, greenish-yellow, ca. 0.3–0.4 mm, truncate, alternate with the petals. *Stamens* 10, in 2 series of 5, either all fertile or all abortive; filaments subulate, whitish-green; anthers orbiculate, pale yellow; fertile stamens with filaments 1.5–2.8 mm and anthers (0.3–)0.4–0.5 mm; abortive stamens with filaments 0.2–0.6 mm and anthers 0.1–0.3 mm. *Ovary* superior; placentation shortly free-central; ovules usually 12 per ovary. *Styles* 3, distinct, erect to ascending; functionally male flowers with styles 0.6–0.9 mm and stigmas scarcely developed; functionally female flowers with styles 1.1–2.1 mm and stigmas linear, glandular-puberulent adaxially. *Capsules* light green to greenish-tan (valve margins tan), on stipe ca. 0.1–0.2 mm, ovoid-conical, 1.8–2.6 × 1.1–1.8 mm slightly longer than (rarely slightly shorter than) and mostly enclosed by the appressed sepals and withering-persistent petals, dehiscing in upper half by 3 valves, these becoming incurved on margins and slightly recurved at tip. *Seeds* apparently 8 per capsule, 0.7–0.8 mm, reddish-black, obliquely reniform with radicle prolonged into a curved bump, somewhat compressed, surfaces sculpted with low bumps at > 10× magnification.

##### Additional specimens examined.

**U.S.A. Washington, Whatcom Co.**: Twin Sisters Range, 11 Aug 1939, *W.C. Muenscher 10281* (WTU); Twin Sisters Range, 12 Aug 1939, *W.C. Muenscher 10306* (WTU); Head of Orsina Creek, at west base of Twin Sisters Mountain, 4900 ft, T37N R6E S11, 12 Jul 1961, *A.R. Kruckeberg 5225* (WTU); Northwest slope of Twin Sisters, ca. 6200 ft, 28 Jul 1968, *R.J. Taylor 2158* (WWB); Crest of ridge along Sisters Divide 0.7 air km southeast of outlet of Lake Wiseman, Twin Sisters Range, 48.704998°N, 121.931408°W; 1508 m, 7 Aug 2016, *B.S. Legler 14268* (ID, US, WTU).

##### Etymology.

The epithet *sororia* is from the Latin word *sororis*, sister, in reference to the Twin Sisters Range.

##### Vernacular name.

Twin Sisters sandwort.

##### Distribution and ecology.

*Sabulina sororia* is known only from the Twin Sisters Range on the western flank of the Cascade Mountains in Whatcom County, Washington, U.S.A. (Fig. 6D). The Twin Sisters Range consists of a large body of relatively unaltered dunite rock aproximately 16 km long by 6.5 km wide (Tabor et al. 2003) oriented in a northwest to southeast direction, with a maximum elevation of 2135 meters and sustained ridgeline elevations above 1500 meters. The dunite rock likely formed in the earth’s mantle and was subsequently uplifted along a series of nearly vertical thrust faults (Ragan 1963); it is a dense, crystalline, ultramafic rock composed mostly of olivine with lesser amounts of chromite and pyroxenite, rich in magnesium, iron, chromium and nickel (Ragan 1963, Onyeagocha 1978). The rock weathers to a distinctive light reddish-brown color with a coarse-grained surface. Ultramafic rocks display a pronounced effect on the overlying vegetation (Kruckeberg 2002, and references therein), and the Twin Sisters dunite is no exception with its depressed treeline and sparse vegetation cover above treeline.

*Sabulina sororia* is apparently restricted to rocky or gravelly, sparsely vegetated, subalpine and alpine slopes. Documented elevations range from 1490 to 1890 meters. Habitat information from older herbarium specimens is sparse, indicating only a “west-facing alpine ridgeline” (*Grable 5023*), “moist, gravelly, serpentine soil on an alpine slope” (*Taylor 2158*), “along streambank” (*Muenscher 10281*), and “olivine in massive fell-fields and talus, with krummholz lodgepole pine and subalpine fir in snow-melt basin” (*Kruckeberg 5225*). At the two sites visited by B. Legler in August 2016, *S. sororia* was observed growing most frequently in mesic, coarse, gravelly and rocky soil derived from dunite on erosional surfaces with slopes ranging from flat to ca. 30° (Fig. 5D–F). A few plants were found in exposed crevices of stable dunite rock outcrops along a narrow ridgeline with slopes of ca. 45–60°. The species apparently avoids areas with late-lying snow. *Sabulina sororia* occurs as scattered individuals, forming a minor component of the sparse, low vegetation cover. Total vegetation cover of all plant species at these two sites is estimated at 5–20%. Average precipitation for the higher elevations of the Twin Sisters Range is estimated at ca. 180–190 cm per year, with about 30% of the total precipitation falling during May–September (PRISM 2017).

Directly associated species consist of scattered tufts or mats of *Carex spectabilis* Dewey, *Cassiope mertensiana* (Bong.) G. Don, Cerastium arvense L. subsp. strictum Gaudin, *Cryptogramma acrostichoides* R. Br., *Danthonia intermedia* Vasey, *Erigeron aureus* Greene, *Polystichum lemmonii* Underw., *Sabulina rubella* (Wahlenb.) Dillenb. & Kadereit, *Saxifraga cespitosa* L., *Sibbaldia procumbens* L., *Silene acaulis* (L.) Jacq., and *Smelowskia ovalis* Rydb. Trees and taller shrubs are absent from these sites, though adjacent ridgelines and slopes hold patches of *Abies lasiocarpa* (Hook.) Nutt., *Callitropsis nootkatensis* (D. Don) D.P. Little, Pinus contorta Douglas ex Loudon var. latifolia Engelm., *Tsuga mertensiana* (Bong.) Carrière, Juniperus communis L. var. kelleyi R.P. Adams, and *Phyllodoce empetriformis* (Sm.) D. Don. Crustose lichens are sparse, and bryophytes nearly absent.

The southern terminus of the Twin Sisters Range extends slightly into adjacent Skagit County, and 6 km farther to the southeast of this are two smaller dunite bodies exposed at slightly lower elevation (Tabor et al. 2003). An examination of aerial imagery suggests marginally suitable habitat for *Sabulina sororia* may occur in these areas, though no surveys have been conducted to determine its presence. It seems unlikely that *S. sororia* will be found elsewhere in the Cascades Mountains or over non-dunite rocks; however, small, subalpine exposures of ultramaphic rocks in Skagit and Snohomish counties may warrant investigation.

##### Phenology.

Specimens indicate the flowering period for *Sabulina sororia* extends from mid July to mid August, and fruiting period from early to mid August. The full ranges of flowering and fruiting periods likely vary based on timing of snowmelt and site exposure.

##### Conservation status.

Although apparently restricted to the Twin Sisters Range, *Sabulina sororia* may occur in suitable microsites throughout the upper elevations of the range within an extent of occurrence estimated at ca. 16 km^2^. The total number of plants cannot be estimated due to inadequate sampling across the range, possibly preventing assignment of a formal conservation status at this time. The Twin Sisters Range lies almost fully within the Mt. Baker-Snoqualmie National Forest, and the entire northeastern slope of the range lies within the Mt. Baker Wilderness. No roads or trails penetrate the range, resource extraction is absent from the higher elevations, and very few people visit each year due to difficulty of access. Direct anthropogenic impacts are therefore assumed to be very minimal.

**Figure 4.**
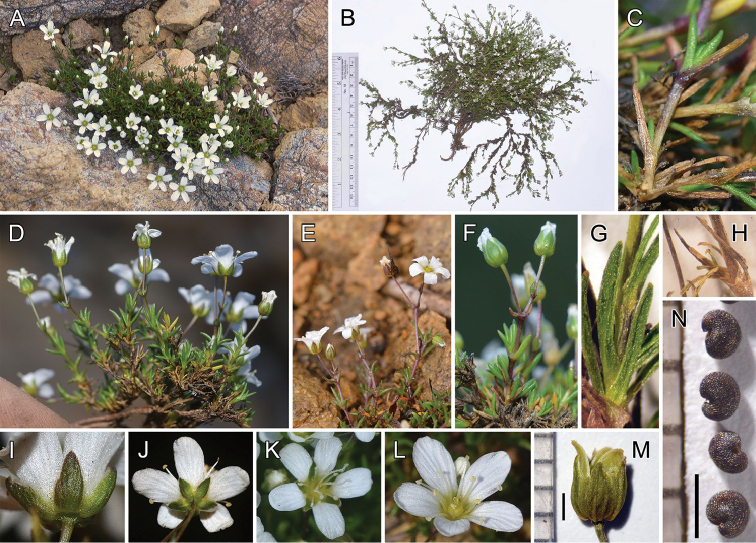
*Sabulina sororia*. **A** Plant forming a loose mat (*Legler 14263*) **B** Excavated plant with loosely sprawling stems (*Legler 14268*) **C** Fresh leaves and persisting, 1-veined, dead leaves (*Legler 14263*) **D** Excavated plant (*Legler 14263*) **E–F** Cymose inflorescences (*Legler 14263*) **G** Dried, 1-veined leaves (*Legler 14263*) **H** Dead leaves with only midvein persisting (*Legler 14263*) **I** Ovate-lanceolate, purple-tinged sepals (*Legler 14263*) **J** Sepals and petals, showing shapes and relative lengths (*Legler 14263*) **K–L** Flowers with different combinations of stamen and style lengths (*Legler 14263*) **M** Dried flower with dehisced capsule longer than sepals (*Legler 14263*) **N** Seeds (*Legler 14263*). Black scale bars are 1 mm.

**Figure 5.**
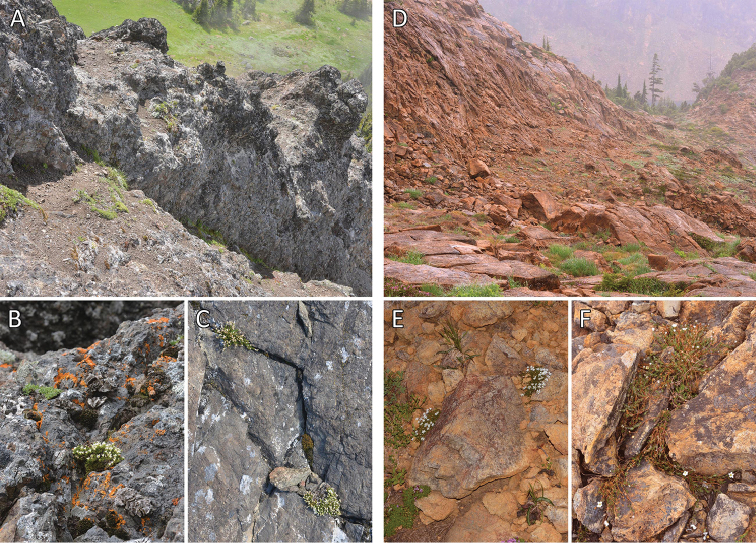
Representative habitats. **A–C** *Sabulina basaltica* habitat **A** Basalt slope near the type locality (*Legler 14177*) **B–C** Plants in crevices of basalt rock faces (*Legler 14177*, *Legler 14183*) **D–F** *Sabulina sororia* habitat **D** Reddish-colored dunite slope at the type locality (*Legler 14263*) **E–F** Plants among dunite rock and gravel (*Legler 14263*, *Legler 14268*).

## Discussion

**Figure 6.**
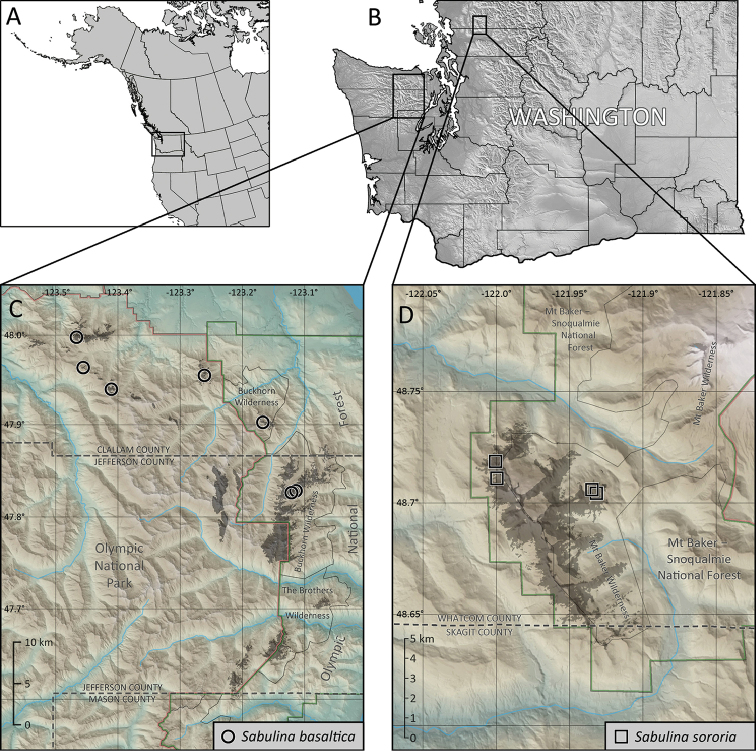
Distribution of *Sabulina basaltica* and *S. sororia*. **A–B** Reference maps of western North America and Washington State, indicating locations of inset maps **C** Known locations for *Sabulina basaltica* (open black circles) within the northeastern Olympic Mountains; gray shading indicates the predicted extent of potential habitat based on exposures of oceanic basalt rocks at subalpine to alpine elevations **D** Known locations for *Sabulina sororia* (open black squares) within the Twin Sisters Range; gray shading indicates the predicted extent of potential habitat based on exposures of dunite rock away from large snowfields at subalpine to alpine elevations.

*Sabulina basaltica* and *S. sororia* can be reliably distinguished from each other morphologically (Table 1, and see key), with the differences comparable to those used to distinguish among other members of the *S. rossii* species complex (e.g., leaf length and veination, sepal length and shape, petal length relative to sepals, capsule length, and seed size and color), suggesting they are appropriately recognized at the same taxonomic rank of species. Their recognition as two distinct species is further supported by the absence of plants with intermediate morphology that could not be unambiguously assigned, their disjunct geographic distributions relative to each other (Fig. 6) and all other glabrous, perennial *Sabulina* species, and their unique ecological niches. Attempts to re-circumscribe any of the previously published *Sabulina* taxa to accommodate *S. basaltica* or *S. sororia* would be impractical.

The results of the molecular phylogenetic analyses (Fig. 1) also support the existence of two independent taxa in Washington. *Sabulina sororia* is part of a group of four closely species (*S. rossii*, *S. elegans*, *S. austromontana* and *S. sororia*), while *S. basaltica* is outside of this group (Fig. 1A). In the ITS phylogeny (Fig. 1A), all species are either supported as monophyletic, or the samples are in a polytomy with samples of one or more other species of the *S. rossii* species complex. The two samples of *S. basaltica* are not closely related to other sequenced samples of the complex, but are supported as sister to each other. Given the general pattern, the lack of affinity of *S. basaltica* to other samples of the *S. rossii* species complex supports the status of *S. basaltica* as an independent species.

ITS sequence data point to an unexpectedly close relationship between *S. sororia* and *S. austromontana* (Fig. 1A) even though they are readily distinguished morphologically (Table 1). The close relationship may indicate that growth form, inflorescence architecture and petal length are labile within the *S. rossii* species complex. An analogous situation is found within the European genus *Facchinia* Rchb., recently split out of *Minuartia* s. lat. by Dillenberger and Kadereit (2014). In that study, *F. cherlerioides* (Sieber) Dillenb. & Kadereit and *F. grignensis* (Rchb.) Dillenb. & Kadereit were unexpectedly resolved as sister species by ITS sequence data, yet they differ substantially from each other in morphology (Dillenberger and Kadereit 2015).

*Sabulina basaltica* and *S. sororia* resemble, and might be confused with, *S. macrantha* and forms of *S. stricta* found in the southern Rocky Mountains and California, based on shared characters of cymose inflorescences and a similar low growth form with relatively short pedicels. Both of the new species differ from *S. macrantha* by their shorter leaves, shorter sepals, shorter capsules, and smaller seeds with less rugose surfaces, and from *S. stricta* in their markedly longer petals, shorter capsules, and larger, dark reddish-brown to blackish seeds. *Sabulina basaltica* further differs from *S. macrantha* and *S. stricta* in its strongly 3-veined leaves.

Hitchcock et al. (1964) treated both *Sabulina basaltica* and *S. sororia* as Arenaria rossii var. rossii (= *S. rossii*). This questionable application of name may reflect Hitchcock’s preference for conservative, or broad, species concepts (Hitchcock et al. 1955). *Sabulina rossii* is distinguished from both of the new species and all other glabrous, perennial *Sabulina* species in North America by the presence of vegetative propagules formed from tight, readily dislodged axillary or terminal leaf fascicles (in other species the fascicles remain firmly attached). The main stem leaves of *S. rossii* are also typically spreading (vs. ascending) and strongly fleshy with a fleshy sheath, usually giving them the appearance of being connate-perfoliate. The sepals of *S. rossii* are 1-veined, while both *S. basaltica* and *S. sororia* have 3-veined sepals, and flowers in *S. rossii* are strictly solitary (vs. partially cymose). *Sabulina rossii* is restricted to the high arctic, mostly in areas near the Arctic Ocean and adjacent connected waterbodies.

*Sabulina rubella* (Wahlenb.) Dillenb. & Kadereit, a normally stipitate-glandular species which also occurs in the Cascade and Olympic mountains, very rarely produces glabrous individuals (Rabeler et al. 2005) with a low, tufted growth form and short pedicels (B. Legler, pers. obs.). These plants may be separated from *S. basaltica* and *S. sororia* by their larger capsules (4–4.5 mm), smaller (0.4–0.5 mm), reddish-brown seeds, broadly ovate to elliptic petals abruptly narrowed to the clawed base, and acute to subulate leaf tips. The authors know of no such glabrous plants of *S. rubella* from Washington State.

The close relationship between *Sabulina fontinalis* and the *S. rossii* species complex (Fig. 1) has been noted in other studies (Greenberg and Donoghue 2011, Dillenberger and Kadereit 2014). Here we exclude *S. fontinalis* from the *S. rossii* species complex due to its divergent morphology (e.g., plants annual; stems square in cross-section; leaves to 4 mm wide; flowers mostly 4-merous) which has led previous authors to place *S. fontinalis* variously under *Sagina* L., *Spergula* L., or *Stellaria* L. (e.g., Rabeler et al. 2005).

Several of the characters commonly used to distinguish among glabrous, perennial *Sabulina* taxa in North America warrant further clarification. Leaf veins are usually not visible in fresh material, but upon drying or decaying show a single prominent midvein and, in some species, a faint to prominent pair of lateral veins. The inflorescence may consist solely of solitary, terminal flowers not subtended by bracts, or partly to fully of 2–many-flowered, bracteate cymes. However, in several species, including *S. basaltica* and *S. sororia*, one must sometimes use care to look for the 2–several-flowered cymes mixed among the often more numerous solitary flowers. The bracts of cymose inflorescences can usually be distinguished from vegetative leaves by their shorter, proportionately broader shape and thin, scarious margins. Petal length must be used with caution on dried specimens and it should be noted that published descriptions (Maguire 1958, Wolf et al. 1979, Rabeler et al. 2005) appear to under-represent the range of petal lengths for *S. elegans*, *S. michauxii*, and *S. rossii* (Table 1).

The following key includes all glabrous, perennial species of *Sabulina* in North America. Leaf veins should be observed on dried or dead, persisting leaves. Leaf length is for main stem leaves, not axillary fascicles. Sepal veins are often weakly visible on living plants, but become clearly defined on dried specimens. Sepal length is taken at anthesis, as sepals often elongate slightly in fruit. Petal length is for fresh material, and petals may shrink relative to the sepals upon drying; this can be mitigated by carefully arranging and pressing individual flowers between tissue paper.

**Table:** 

1	Plants reproducing vegetatively by means of tight, readily dislodged, axillary or terminal fascicles of leaves; primary stem leaves mostly widely spreading, strongly fleshy and ± connate-perfoliate, often purple-tinged, 1-veined, 1–4 mm; flowers often absent; sepals 1.5–2.5 mm, oblong-ovate, weakly 1-veined, purplish; petals 1.2–2 times as long as sepals, or sometimes < sepals or absent; high arctic of eastern Siberia to North America, Greenland, and Spitzbergen	***Sabulina rossii***
–	Plants not reproducing by means of readily dislodged vegetative propagules; leaves mostly ascending to appressed, with a scarious to herbaceous sheath, 1- or 3-veined; flowers nearly always produced; sepals and petals various	**2**
2	Petals mostly 1.2–2(–2.5) times as long as the sepals; flowers partly in 2–8(–30)-flowered, terminal cymes (often also some flowers solitary and terminal, rarely all flowers solitary and terminal)	**3**
–	Petals 0.5–1(–1.1) times as long as the sepals, or rudimentary to absent; flowers partly in 2–5(–8)-flowered, terminal cymes, or flowers all solitary and terminal	**6**
3	Sepals 3–6 mm; main stem leaves 5–30 mm; shoots of current year’s growth 2–40 cm; seeds 0.8–1.1 mm, blackish	**4**
–	Sepals 1.4–2.8(–3.3) mm; main stem leaves 0.6–4(–4.5) mm; shoots of current year’s growth 0.5–4 cm; seeds 0.6–0.8 mm, dark reddish-brown to reddish-black	**5**
4	Inflorescences 5–30-flowered; longer pedicels gen > 15 mm; stems erect to ascending, 8–40 cm; leaves 8–30 mm, tips blunt to pungent; petals 1.3–2 times as long as sepals (or < sepals in northern plants); Great Plains to northeast U.S.A. and southeast Canada	***Sabulina michauxii***
–	Inflorescences 1–5(–8)-flowered; longer pedicels 3–15(–20) mm; stems procumbent to ascending, 2–15 cm; leaves 5–10 mm, tips rounded; petals 0.7–1.8 × as long as sepals; Rocky Mountains from Wyoming to New Mexico, west to Nevada	***Sabulina macrantha***
5	Leaves 3-veined; sepals (1.6–)2.4–2.8(–3.3) mm, lanceolate to narrowly ovate-lanceolate, (2.4–)3–3.2(–3.5) times as long as wide; mature capsules mostly slightly < sepals; pedicels 1–3.5(–6) mm; plants usually forming dense, tight mats or cushions; crevices of exposed basalt summits; Olympic Mountains, Washington	***Sabulina basaltica***
–	Leaves 1-veined; sepals (1.4–)1.7–2.5(–3) mm, ovate to ovate-lanceolate, mostly 1.5–2.5 times as long as wide; mature capsules mostly > sepals; pedicels (1–)2–8(–15) mm; plants mat-forming or trailing; bare dunite rock and gravel; Twin Sisters Range, Washington	***Sabulina sororia***
6	Flowers all solitary and terminal at stem tips, not subtended by bracts (upper-most stem leaves not distinct from those below); leaves 1-veined	**7**
–	Flowers partly in 2–15-flowered, bracteate, terminal cymes (often also some flowers solitary and terminal, very rarely all flowers solitary and terminal); bracts generally with thin, scarious margins, often smaller and broader than the stem leaves; leaves 1-veined or weakly 3-veined	**8**
7	Sepals light green, linear-lanceolate to narrowly lanceolate; petals absent or rudimentary (rarely nearly equaling sepals); pedicels 3–15(–20) mm; plants tightly cespitose; Rocky Mountains of southern Canada and northern U.S.A.	***Sabulina austromontana***
–	Sepals purplish, lanceolate to ovate-lanceolate; petals usually present (occasionally absent), 0.6–1(–1.1) times as long as sepals; pedicels 10–40 mm; plants loosely tufted to cespitose; eastern Siberia, Alaska, and northwest Canada to Rocky Mountains of central Canada	***Sabulina elegans***
8	Seeds 0.7–1.1 mm, blackish, prominently rugose at 10 × magnification; sepals 3–6 mm at anthesis; southeast Canada and eastern U.S.A., or U.S.A. Rocky Mountains	**see couplet 4**
–	Seeds 0.4–0.6 mm, reddish-brown to blackish, obscurely rugose at 10 × magnification; sepals (1.5–)2–4 mm at anthesis	**9**
9	Inflorescences (2–)7–15-flowered; capsules 3.5–4.5 mm, equaling or longer than sepals; seeds dark brown to blackish; sepals ovate to lanceolate; stems ascending to erect, 4–30 cm; Alaska and much of Canada, south to north-central U.S.A.	***Sabulina dawsonensis***
-	Inflorescences 1–3(–5)-flowered; capsules 2.5–3.2 mm, equaling or shorter than sepals; seeds reddish-brown to brown; sepals elliptic-ovate to ovate-lanceolate; stems decumbent to erect, 0.8–12 cm; circumboreal in arctic regions, and disjunct in Colorado and California	***Sabulina stricta***

*Sabulina basaltica* joins seven other vascular plant taxa endemic to higher elevations of the northeastern and eastern portions of the Olympic Mountains: Astragalus australis (L.) Lam. var. cottonii (M.E. Jones) S.L. Welsh, *Campanula piperi*, *Erigeron flettii* G.N. Jones, *Petrophytum hendersonii*, *Senecio neowebsteri* S.F. Blake, *Synthyris lanuginosa* (Piper) Pennell & J.W. Thomp., and *Viola flettii*. These are all concentrated on relatively dry, subalpine to alpine rock faces, scree slopes and tundra-like meadows and co-occur with several plant taxa widely disjunct from the Rocky Mountains, such as *Astragalus microcystis* A. Gray, *Carex obtusata* Lilj., and Oxytropis borealis DC. var. viscida (Nutt.) S.L. Welsh. In general, the vascular plant flora of the Olympic Mountains shows relatively high levels of endemism and floristic similarities to both the Rocky Mountains and coastal and boreal regions of British Columbia and Alaska (Houston et al. 1994, Buckingham et al. 1995). The mountains are disconnected from other high-elevation ranges by encircling lowlands and saltwater.

To explain these patterns it is widely proposed that the northeastern Olympic Mountains acted as a refugium during Pleistocene glacial advances (Buckingham et al. 1995, Houston et al. 1994, Peterson et al. 1997, Gavin et al. 2013). The Cordilleran ice sheet reached a maximum depth of ca. 1000 meters in the lowlands along the northern and eastern sides of the mountains ca. 17,000 yr B.P. (Porter and Swanson 1998), leaving ridgelines well above the ice, while alpine glaciers were concurrently limited by relatively arid regional conditions (Thackray 2001) and had furthermore began retreating from their maximum extent ca. 20,000 yr B.P. (Booth 1987, Thackray 2001). The asynchronous timing of alpine and continental ice undoubtedly led to persistently ice-free areas at high elevations. These areas lie in the rainshadow cast by the bulk of the mountains to the west and remained relatively arid throughout the last glacial maximum and up through the present day (Gavin et al. 2013), potentially providing long-term habitat stability for taxa adapted to dry alpine conditions. As noted by Gavin et al. (2013) and Houston et al. (1994), it is probably no coincidence that the endemic plant taxa in the northeastern Olympics are mostly restricted to the very ridgelines that remained ice-free and relatively dry. *Sabulina basaltica*, likewise adapted to these habitats, may have persisted here through multiple glacial cycles. The lack of a clear close relationship between *S. basaltica* and any other single member of the *S. rossii* species complex (Fig. 1A) provides further evidence of long-term isolation, suggesting *S. basaltica* is a paleoendemic.

In contrast, the Twin Sisters Range, where *Sabulina sororia* is endemic, exhibits relatively low species diversity and was previously thought to house no endemic vascular plant taxa (Kruckeberg 2002). This contrasts with relatively high levels of endemism observed in other large exposures of ultramaphic rocks in western North America, including the Wenatchee Mountains in central Washington and the Klamath-Siskiyou region of southwest Oregon and northeast California (Kruckeberg 2002). However, these latter areas lie south of the Cordilleran ice sheet limits and only experienced alpine glaciation at most. The Twin Sisters Range, by comparison, was enveloped on all sides by an ice sheet estimated at its maximum to be ca. 1500–1800 meters deep in the adjacent lowlands to the west (Porter and Swanson 1998) and reaching elevations of over 2000 meters in the adjacent North Cascade Mountains to the east (Kovanen and Easterbrook 2001), with few peaks apparently remaining above the ice. Observations of non-dunite glacial erratics at just over 1500 m elev. on the Twin Sisters Range (Ragan 1962, Kruckeberg 1991) corroborate these estimates. However, the highest peaks in the range, including North Twin (2012 m) and South Twin (2135 m), reportedly remained above the ice sheet (Ragan 1962), creating a potential refugium. We suspect that *S. sororia*, able to grow on exposed, rocky ridgelines and slopes, may have found suitable habitat within this small refugium to persist in-situ through the last glacial maximum. Alternatively, *S. sororia* may be a neoendemic that colonized and differentiated following the most recent retreat of the ice sheet, as suggested by its close relationship to *S. austromontana* (Fig. 1A). Phylogeographic methods may provide means of testing these scenarios.

## Supplementary Material

XML Treatment for Sabulina basaltica

XML Treatment for Sabulina sororia

## Supplementary materials

Supplementary material 1 Voucher information for herbarium specimens examined Data type: occurrence Explanation note: General information and provenance for herbarium vouchers of *Sabulina* taxa examined for morphological characters and geographic distribution. Included are taxon names, general collection locations, collector names and numbers, herbarium acronyms and accession numbers. This dataset is made available under the Open Database License (http://opendatacommons.org/licenses/odbl/1.0/). The Open Database License (ODbL) is a license agreement intended to allow users to freely share, modify, and use this Dataset while maintaining this same freedom for others, provided that the original source and author(s) are credited.Ben S. Legler, Markus S. Dillenberger

Supplementary material 2 Sample information and GenBank accessions of sequenced specimens Data type: occurrence Explanation note: Voucher specimens of Caryophyllaceae taxa sequenced for phylogenetic analysis. Included are taxon names, sample extraction numbers, general collection location, collector names and numbers, herbarium acronyms and accession numbers, and GenBank numbers for ITS and trnQ-rps16 sequences. This dataset is made available under the Open Database License (http://opendatacommons.org/licenses/odbl/1.0/). The Open Database License (ODbL) is a license agreement intended to allow users to freely share, modify, and use this Dataset while maintaining this same freedom for others, provided that the original source and author(s) are credited.Ben S. Legler, Markus S. Dillenberger
