# Supplementary material for: Two new species of Sabulina (Caryophyllaceae) from Washington State, U.S.A
Source: PhytoKeys. 2017 Jun 15;(81):79–102. doi: 10.3897/phytokeys.81.13106 (PMC5523872; doi:10.3897/phytokeys.81.13106)
Supplement: Supplementary material 1 — Voucher information for herbarium specimens examined [file phytokeys-81-079-s001.pdf]

**Appendix 1.** Voucher information for 127 herbarium specimens examined.

Taxon: country, state/province, county: collector coll. # (herbarium acronym and no.);

***Sabulina austromontana*** (S.J. Wolf & Packer) Dillenb. & Kadereit: Canada, Alberta: *K.I. Beamish 1978* (UBC V163168); British Columbia: *G.W. Douglas & J.L. Penny 13130* (V 167438); *H. Roemer 96268* (V 168573); U.S.A., Idaho, Custer Co.: *C.L. Hitchcock & C.V. Muhlick 10951* (WTU 94489); Montana, Beaverhead Co.: *C.L. Hitchcock & C.V. Muhlick 12860* (WTU 100468); *C.L. Hitchcock & C.V. Muhlick 12908* (WTU 100469); Carbon Co.: *A. Cronquist 8003* (WTU 174049); Fergus Co.: *P. Lesica 1662* (MONTU 87364); *C.L. Hitchcock 12037* (WTU 100471); Glacier Co.: *P. Lesica 5575* (MONTU 115031); *L.H. Harvey & R.H. Pemble 7146* (WTU 231297); *L.H. Harvey & R.H. Pemble 7127* (WTU 231298); Granite Co.: *K.H. Lackschewitz 6181* (WTU 266600); Madison Co.: *P. Lesica 7930* (MONTU 125482); Powell Co.: *C.L. Hitchcock 18624* (WTU 126459); Stillwater Co.: *P. Lesica 7673* (MONTU 122803); Wyoming, Park Co.: *C.L. Porter 5855* (WTU 148737). ***Sabulina basaltica*** B.S. Legler: U.S.A., Washington, Clallam Co.: *M.P. Harthill s.n.* (OLYM 4262); [*collector not indicated*] (OLYM 4263); *N. Buckingham s.n.* (OLYM 16642); *E.L. Tisch s.n.* (OLYM 38389); U.S.A., *E.L. Tisch s.n.* (OLYM 38390); *G.N. Jones 3202* (WTU 21910); *H.E. Helmrich 259* (WTU 306174); *L.C. Bliss s.n.* (WTU 337168); *B.S. Legler 14184* (WTU 414404); *B.S. Legler 14183* (WTU 414405); *B.S. Legler 14179* (WTU 414406); *B.S. Legler 14178* (WTU 414407); *B.S. Legler 14177* (WTU 414408); *J.W. Thompson 5481* (WTU 19406JWT); *J.W. Thompson 9458* (WTU 19406JWT); *J.W. Thompson 7433* (WTU 19407JWT); Jefferson Co.: *N. Buckingham s.n.* (OLYM 4260); *B.S. Legler 14195* (WTU 414403); *J.W. Thompson 11054* (WTU 7814JWT). ***Sabulina dawsonensis*** (Britton) Rydb.: Canada, Alberta: *M.O. Malte & W.R. Watson 1576* (WTU 223724); British Columbia: *F. Lomer 5779* (UBC V233413); *R. Hebda & R. Forsyth KM5001* (V 190228); *A. Ceska 7612* (V 193884); Manitoba: *W.B. Schofield & H.A. Crum 6693* (WTU 211362); *W.B. Schofield & H.A. Crum 7125* (WTU 211363); *W.B. Schofield & H.A. Crum 6594* (WTU 211368); Yukon Territory: *B. Bennett 03/0176* (ALA H1132189); *W.B. Schofield & H.A. Crum 7544* (WTU 226284); U.S.A., Alaska: *C.L. Parker & J. Herriges 17201* (ALA H1106040); *B. Bennett 03-253* (ALA H1132193); *C.L. Parker 2725* (ALA H1132201); *C.L. Parker & M. Gracz 6883* (ALA H1132205). ***Sabulina elegans*** (Cham. & Schltdl.) Dillenb. & Kadereit: Canada, British Columbia: *R. Revel s.n.* (UBC V168182); *G.W. Argus & E. Haber 9959* (V 114959); *K.L. Marr, R. Hebda & W. MacKenzie 04-1229* (V 196333); *K.L. Marr 09-0610* (V 205312); Northwest Territories: *H.M. Raup & J.H. Soper 9655* (UBC V90295); Yukon Territory: *D.F. Murray 1575* (ALA H1132248); U.S.A., Alaska: *G.N. Jones 9070* (WTU 37227); *G.N. Jones 9070* (WTU 12039JWT); *S. Studebaker 07-122* (ALA H1063909); *C.L. Parker 15665* (ALA H1132230); *A. Larson 02-1770b* (ALA H1132273); *C.L. Parker 711* (ALA H1132293); *D. R. Hunt 30B* (ALA H1132314); *R. Lipkin 84-30* (ALA H1132315); *C.L. Parker, A.R. Batten, M. Duffy & J. Cole 7329* (ALA H1132316); *C.L. Parker 7579* (ALA H1132317); *C.L. Parker, M. Duffy & D. Blank 7167* (ALA H1132318); *A.R. Batten 96-310* (ALA H1132368); *C.L. Parker & S. Studebaker 16154* (ALA H1132387); *C.L. Parker & C.R. Meyers 10561* (ALA H1132698); *C.L. Parker, A.R. Batten & L. Jeschke 10451* (ALA H1132701); *D. Killdaw s.n.* (ALA H1132712); *J. Ebersole 266* (ALA H1133039); *C.L. Parker 3512* (ALA H1133046); *I.L. Wiggins 13653* (WTU 178194); *G.H. Ward 1169* (WTU 193132); *I.L. Wiggins 12658* (WTU 193193); *M. Williams 2378* (WTU 254390); *V. Komarkova, H. Hansell & K. Seabert 387* (WTU 271663). ***Sabulina macrantha*** (Rydb.) Dillenb. & Kadereit:U.S.A., Arizona, Coconino Co.: *P.A. Munz 16977* (WTU 169748); Colorado, Garfield Co.: *J.L. Wingate 10508* (KHD KHD00061331); Gunnison Co.: *E.L. Hartman 6220* (KHD KHD00019416); *W.A. Weber & P. Dixon 16341* (WTU 288061); Park Co.: *E.L. Hartman 6357* (KHD KHD00019424); *W.A. Weber 8751* (WTU 163935); San Juan Co.: *E.L. Hartman 5097* (KHD KHD00016803); Nevada, Clark Co.: *I.W. Clokey 7923* (WTU 65384). ***Sabulina michauxii*** (Fenzl) Dillenb. & Kadereit: Canada, Ontario: *T.M.C. Taylor, M.W. Bannan & H.M.*

*Harrison 255* (WTU 192664); *F. Marie-Victorin, F. Rollland-Germain & F. Dominique 46461* (WTU 11851JWT); Quebec: *M.L. Fernald & C.A. Weatherby 547* (WTU 61133); U.S.A., Indiana: *L.M. Umbach 3340* (WTU 6861); *W.S. Moffatt s.n.* (WTU 95381); Indiana, Lake Co.: *W.S. Moffatt s.n.* (WTU 6871); *H.R. Bennett s.n.* (WTU 178003); Massachusetts, Franklin Co.: *H.E. Ahles 86703* (WTU 281539); Michigan: *F.H. Burglehaus s.n.* (WTU 6816); *W.E. Mulliken s.n.* (WTU 20529); Missouri: *F. Comte 2188* (WTU 319179); New York: *H.H. Keyes s.n.* (WTU 95383); Oklahoma: *G.M. Merrill & T.A. Hagen 249* (WTU 6235JWT); Pennsylvania, Bedford Co.: *D. Berkheimer 11165* (WTU 162629); Vermont: *D. L. Hutton s.n.* (WTU 44400); West Virginia, Grant Co.: *R. Burton s.n.* (WTU 77507); Mr. & Mrs. *H.A. Davis 7844* (WTU 107768). ***Sabulina rossii*** (R. Br. ex Richardson) Dillenb. & Kadereit: Canada, Northwest Territories/Nunavut: *R. Elvan 3253* (ALA H1132737); Nunavut: *W.A. Gould s.n.* (ALA H1132741); Yukon Territory: *J.A. Parmelee 2775a* (UBC V149598); Russia, Chukotka: *H. Solstad 05/0448* (ALA H1046947); U.S.A., Alaska: *John Putnam 33* (WTU 261326); *L. Viereck 4289* (ALA H1132707); *R.L. Rausch s.n.* (ALA H1132726). ***Sabulina sororia*** B.S. Legler: U.S.A., Washington, Whatcom Co.: *W.C. Muenscher 10281* (WTU 56018); *W.C. Muenscher 10306* (WTU 56019); *A.R. Kruckeberg 5225* (WTU 216592); *B.S. Legler 14263* (WTU 414409); *B.S. Legler 14268* (WTU 414410); U.S.A., *R.J. Taylor 2158* (WWB 11914). ***Sabulina stricta*** (Sw.) Rchb.: Canada, Manitoba: *W.B. Schofield & H.A. Crum 7033* (WTU 211364); *W.B. Schofield & H.A. Crum 6836* (WTU 211369); Yukon Territory: *D.F. Murray 1740* (ALA H1133034); *D.F. Murray 1789* (ALA H1133035); Greenland: *C. Bay 629* (ALA H1133059); Sweden, Lappland: *G. Samuelsson 763* (ALA H1133060); U.S.A., Alaska: *C. L. Parker, R. Elvan & H. Solstad 15114* (ALA H1133043); *I.L. Wiggins 13796* (WTU 175814); Colorado, Hinsdale Co.: *E.L. Hartman 5139* (KHD KHD00019422); Park Co.: *E.L. Hartman 5631A* (KHD KHD00019420); *W.A. Weber 6551* (WTU 145540).
