# Supplementary material for: Two new species of Sabulina (Caryophyllaceae) from Washington State, U.S.A
Source: PhytoKeys. 2017 Jun 15;(81):79–102. doi: 10.3897/phytokeys.81.13106 (PMC5523872; doi:10.3897/phytokeys.81.13106)
Supplement: Supplementary material 2 — Sample information and GenBank accessions of sequenced specimens [file phytokeys-81-079-s002.pdf]

Appendix 2. Sample information and GenBank accessions of sequenced specimens.

Taxon, extraction no., location, collector coll.# (herbarium acronym and no.), GenBank no. (ITS, trnQ-rps16); *newly published sequences

Colobanthus affinis Hook. f., Min285, New Zealand, South Island, Mt. Goul, *W.R. Barker & al. 890204/46-3* (W 2000-13630), KF737548, *KY700773; ***Colobanthus masonae*** L.B. Moore, Min280, New Zealand, *E. Hörandl & F. Hadacek 7962* (W 1998-03654), KF737555, *KY700774; ***Facchinia herniarioides*** (Rion) Dillenb. & Kadereit, Min272, Switzerland, Valais, Britanniahütte, *M.S. Dillenberger & A.J. Moore 2012191* (MJG 009843), KF737472, *KY700775; ***Facchinia valentina*** (Pau) Dillenb. & Kadereit, Min43, Spain, Comunidad Valenciana, Serra d'Espadá, *M.S. Dillenberger 2307-1* (MJG 004132), KF737543, *KY700776; ***Sabulina attica*** (Boiss. & Sprun.) Dillenb. & Kadereit, Min171, Italy, Posta Manganaro, *R.P. Wagensommer s.n.* (MJG 004227), KF737460, *KY700778; ***Sabulina attica*** (Boiss. & Sprun.) Dillenb. & Kadereit subsp. ***attica***, Min265, Greece, Ioannina, Astraka, *A.J. Richards s.n.* (RNG 2012-7-39), KF737450, *KY700777; ***Sabulina austriaca*** (Jacq.) Rchb., Min6, Austria, Styria, Raxalpe, *A. Tribsch s.n.* (WU), KF737508, *KY700779; ***Sabulina austriaca*** (Jacq.) Rchb., Min81, Austria, Lower Austria, between Terz and Kernhof, *A. Moore 1064* (MJG 005184), KF737443, *KY700780; ***Sabulina austromontana*** (S.J. Wolf & Packer) Dillenb. & Kadereit, Sab4, U.S.A., Montana, Madison County, *Lesica 7930* (MONTU 125482), *KY700759, *KY700781; ***Sabulina austromontana*** (S.J. Wolf & Packer) Dillenb. & Kadereit, Sab5, U.S.A., Montana, Glacier County, *Lesica 5575* (MONTU 115031), *KY700760, *KY700782; ***Sabulina basaltica*** B.S. Legler, Sab1, U.S.A., Washington, Clallam County, *B.S. Legler 14177* (WTU, holotype), *KY700761, *KY700804; ***Sabulina basaltica*** B.S. Legler, Sab3, U.S.A., Washington, Jefferson County, *B.S. Legler 14195* (WTU, paratype), *KY700762, *KY700805; ***Sabulina biebersteinii*** (Rupr.) Dillenb. & Kadereit, Min122, Georgia, Pirikiti Khevsureti District, Dusheti District, *Shetekauri & al. 1113* (W 2006-22657), KF737416, *KY700783; ***Sabulina californica*** (A. Gray) Dillenb. & Kadereit, Min203, U.S.A., California, Oroville, *Ahart 12551 (2006)* (JEPS 110187), KF737453, *KY700784; ***Sabulina cismontana*** (Meinke & Zika) Dillenb. & Kadereit, Min201, U.S.A., California, Oroville, *Ahart 12548 (2006)* (JEPS 110185), KF737456, *KY700785; ***Sabulina dawsonensis*** (Britton) Rydb., Sab6, U.S.A., Alaska, Black River, *Parker 17201* (ALA H1106040), *KY700763, *KY700786; ***Sabulina dawsonensis*** (Britton) Rydb., Sab7, Canada, Yukon Territory, Beaver Creek, *Bennett 03-0176* (ALA H1132189), *KY700764, *KY700787; ***Sabulina douglasii*** (Fenzl ex Torr. & A. Gray) Dillenb. & Kadereit, Min205, U.S.A., California, Paskenta, *Ahart 12,574 (2006)* (JEPS 108819), KF737459, *KY700788; ***Sabulina elegans*** (Cham. & Schltdl.) Dillenb. & Kadereit, Sab8, U.S.A., Alaska, Kodiak Island, *Studebaker 07-122* (ALA H1063909), *KY700765, *KY700789; ***Sabulina elegans*** (Cham. & Schltdl.) Dillenb. & Kadereit, Sab9, U.S.A., Alaska, Ahklun Mountains, *Parker 15665* (ALA H1132230), *KY700766, *KY700790; ***Sabulina foliosa*** (Royle ex Edgew. & Hook. f.) Dillenb. & Kadereit, Min145, Afghanistan, Baghlan, Salang pass, *Fohlen s.n.* (W 2004-01027), KF737427, –; ***Sabulina fontinalis*** (Short & R. Peter) Dillenb. & Kadereit, U.S.A., Tennessee, Trousdale County, *A. McKerrow 93-015* (NY 02497171), JN589045, JN589447; ***Sabulina glaucina*** (Dvořáková) Dillenb. & Kadereit, Min281, Austria, Lower Austria, *T. Barta 825* (W 2012-07412), KF737553, –; ***Sabulina glaucina*** (Dvořáková) Dillenb. & Kadereit, Min170, Romania, Bélavár, *M. Höhn s.n.* (MJG 004127), KF737519, *KY700791; ***Sabulina glaucina*** (Dvořáková) Dillenb. & Kadereit, Min176, Italy, Basilicata, Serra del Prete, *J. Klein Car16.1* (MJG 004228), –, *KY700792; ***Sabulina helmii*** (Fisch. ex. Ser.) Dillenb. & Kadereit, Min268, Russia, Bashkortostan, Ural, *I. M. Krascheninnikov s.n.* (RNG 2017-7-31), –, *KY700793; ***Sabulina juniperina*** (L.) Dillenb. & Kadereit, Min15, Greece, Achaía, Chelmós, *Hörandl & Hadaček 7476* (W), KF737504, –; ***Sabulina kashmirica*** (Edgew. & Hook. f.) Dillenb. & Kadereit, Min114, Botanical Garden Mainz, *M.S. Dillenberger 13024* (MJG 011706), –, *KY700794; ***Sabulina lineata*** (Boiss.) Dillenb. & Kadereit, Min17, Armenia, Ararat, Zangakatun, *Kugler & Vitek 09-0877* (W), KF737507, *KY700795; ***Sabulina macrantha*** (Rydb.) Dillenb. & Kadereit, Sab10, U.S.A., Colorado, San Juan County, *Hartman 5097* (KHD KHD00016803), *KY700767, *KY700796; ***Sabulina macrantha*** (Rydb.) Dillenb. & Kadereit, Sab11, U.S.A., Colorado, Garfield County, *Wingate 10508* (KHD KHD00061331), *KY700768, *KY700797; ***Sabulina mediterranea*** (Ledeb. ex Link) Rchb., Min118, Greece, Kavála, Thasos, *Krendl s.n.* (W 1989-04139),

KF737520, –; ***Sabulina michauxii*** (Fenzl) Dillenb. & Kadereit, Canada, Manitoba, Riding Mountain National Park, *W.J. Cody 24237* (YU 057124), JN589059, JN589450, misidentified as *S. dawsonensis* in Greenberg and Donoghue (2011) and Dillenberger and Kadereit (2014); ***Sabulina nuttallii*** (Pax) Dillenb. & Kadereit var. ***gracilis*** (B.L. Rob.) Dillenb. & Kadereit, Min199, U.S.A., California, Yosemite National Park, *Matson & al. 1511 (2005)* (JEPS 111151), KF737451, *KY700798; ***Sabulina nuttallii*** (Pax) Dillenb. & Kadereit var. ***gregaria*** (A. Heller) Dillenb. & Kadereit, Min198, U.S.A., California, Snow Mts. Wilderness, *Oswald & Ahart 6319 (1994)* (JEPS 95473), KF737446, *KY700799; ***Sabulina pichleri*** (Boiss.) Dillenb. & Kadereit, Min139, Greece, Arkadía, Leonidio, *Burri & Krendl s.n.* (W 1996-10601), KF737418, –; ***Sabulina pusilla*** (S. Watson) Dillenb. & Kadereit, Min207, U.S.A., California, Patterson, *Ertter & McNeal 5782* (JEPS 84958), KF737452, –; ***Sabulina rimarum*** (Boiss. & Balansa) Dillenb. & Kadereit var. ***rimarum***, Min147, Turkey, Goruh, Şavşat, *F. Sorger 81-72-82* (W 1991-00747), KF737514, –; ***Sabulina rosei*** (Maguire & Barneby) Dillenb. & Kadereit, Min204, U.S.A., California, Klamath Mountains, *Taylor 12734 (1992)* (JEPS 090761), KF737476, *KY700800; ***Sabulina rossii*** (R. Br. ex Richardson) Dillenb. & Kadereit, Sab12, Russia, Chukchi Peninsula, *Solstad 05/0448* (ALA H1046947), *KY700769, –; ***Sabulina rossii*** (R. Br. ex Richardson) Dillenb. & Kadereit, Sab13, Canada, Nunavut, Mt. Pelly, *Gould s.n.* (ALA H1132741), *KY700770, *KY700801; ***Sabulina rubella*** (Wahlenb.) Dillenb. & Kadereit, Min195, U.S.A., *Greenhouse 5011 (2000)* (JEPS 97795), KF737481, *KY700802; ***Sabulina rubella*** (Wahlenb.) Dillenb. & Kadereit, Min240, Canada, North-West Territory, Ellesmere Island, *S.P. Thornton-Wood & R. McTeague 182* (RNG 2017-7-79), –, *KY700803; ***Sabulina sororia*** B.S. Legler, Sab2, U.S.A., Washington, Whatcom County, *B.S. Legler 14263* (WTU, holotype), *KY700771, *KY700806; ***Sabulina stolonifera*** (T.W. Nelson & J.P. Nelson) Dillenb. & Kadereit, Min202, U.S.A., California, Scott Mountain, *D.W. Taylor 18120* (2002) (JEPS 100204), KF737447, *KY700807; ***Sabulina stricta*** (Sw.) Rchb., Min206, U.S.A., California, White Mountain Peak, *Morefield & Pollak 4084 (1986)* (UC 1549872), KF737449, *KY700809; ***Sabulina stricta*** (Sw.) Rchb., Sab15, U.S.A., Colorado, Park County, *Hartman 5631A* (KHD KHD00019420), *KY700772, *KY700808; ***Sabulina sublineata*** (Rech. f.) Dillenb. & Kadereit, Min116, Iran, Hamadan, Abbas-abad, Kouhe' Alvand, *Termé & Moussavi 16380* (W 1975-6996), –, *KY700810; ***Sabulina sublineata*** (Rech. f.) Dillenb. & Kadereit, Min136, Iran, Kordestān, Hamzeh Arab, *Lamond & Termé 42570* (W 1996-06574), KF737434, –; ***Sabulina subtilis*** (Fenzl ex Boiss.) Dillenb. & Kadereit, Min107, Iran, Hamadan, Abas Abad, *Assadi & Amini 13605* (E 421745), KF737487, *KY700811; ***Sabulina tenuifolia*** (L.) Rchb., Min11, Greece, Dodekánisos, Karpathos, *Burri & Krendl s.n.* (W 2001-0003917), KF737510, *KY700813; ***Sabulina tenuifolia*** (L.) Rchb., Min12, Austria, Lower Austria, Steinfeld, *Barta s.n.* (W 2000-0009796), KF737505, *KY700814; ***Sabulina tenuifolia*** (L.) Rchb., Min44, Spain, Pais Vasco, Arantzazu, *M.S. Dillenberger 3107-4d* (MJG 004124), KF737417, *KY700815; ***Sabulina tenuifolia*** (L.) Rchb., Min101, Saudi Arabia, Jalal Ad Dafa, *Collenette 8544* (E 118360), –, *KY700812; ***Sabulina thymifolia*** (Sibth. & Sm.) Dillenb. & Kadereit, Min262, Lebanon, North Lebanon, *The Darwin Project 561* (RNG 2012-7-59), KF737435, *KY700816; ***Sabulina thymifolia*** (Sibth. & Sm.) Dillenb. & Kadereit, Min263, Lebanon, Beirut, *The Darwin Project 552* (RNG 2012-7-58), KF737545, *KY700817; ***Sabulina umbellulifera*** (Boiss.) Dillenb. & Kadereit subsp. ***pontica*** (Bornm.) Dillenb. & Kadereit, Min278, Turkey, Sivas, Kunduz Dag, *F. Sorger 69-52-3* (W 1991-00931), KF737550, –; ***Sabulina umbellulifera*** (Boiss.) Dillenb. & Kadereit subsp. ***umbellulifera***, Min277, Turkey, Antalya, Susuzdag, *F. Sorger T-65-24-62* (W 1991-00493), KF737547, –; ***Sabulina velenovskyi*** (Rohlena) Dillenb. & Kadereit, Min173, Macedonia, Ohrid, Galičica Mt., *Klein & Gencheva 2.9.14.3* (MJG 004108), KF737461, *KY700818; ***Sabulina verna*** (L.) Rchb. subsp. ***hercynica*** (Willk.) Dillenb. & Kadereit, Min267, Germany, Lower Saxony, Harz, *G. Gottschlich 9068* (RNG 2012-7-32), KF737477, *KY700819; ***Sabulina verna*** (L.) Rchb. subsp. ***kabylica*** (Pomel) Dillenb. & Kadereit, Min244, Morocco, Marrakech, *S.L. Jury & al. 18913* (RNG 2012-7-73), KF737468, *KY700820; ***Sabulina verna*** (L.) Rchb. subsp. ***verna***, Min154, France, Alpes Maritimes, *Moore & Ichter 1267* (MJG 004018), KF737432, *KY700821; ***Sabulina verna*** (L.) Rchb. subsp. ***verna***, Min286, Italy, Lombardia, Passo di Croce Domini, *M.S. Dillenberger 201278* (MJG 009755), KF737554, –; ***Sabulina villarsii*** (Balb.) Rchb., Min111, Spain, La Masella, Como Oriola, *Nualart & al. s.n.* (BC 864134), –, *KY700822; ***Sabulina villarsii*** (Balb.) Rchb., Min112, Spain, Berguedà, Bagà, *Soriano & Forurs 2703* (BC 865921), KF737421, *KY700823; ***Sabulina viscosa***

(Schreb.) Rchb., Min250, Bulgaria, Sozopol, *OPTIMA ITER IX 551* (RNG 2012-7-13), KF737464, *KY700824; ***Sagina micropetala*** Rauschert, Min209, Germany, Rhineland-Palatinate, Mainz, *M.S. Dillenberger 20121* (MJG 007678), KF737496, *KY700825; ***Sagina procumbens*** L., Min183, Bulgaria, Kyustendil, Rila, *Klein & Gencheva 29.8.7.2* (MJG 004137), KF737444, *KY700826.
